# Direct-from-sputum rapid phenotypic drug susceptibility test for mycobacteria

**DOI:** 10.1371/journal.pone.0238298

**Published:** 2020-08-28

**Authors:** Timothy E. Butler, Aiden J. Lee, Yongqiang Yang, Mitchell D. Newton, Roli Kargupta, Sachidevi Puttaswamy, Shramik Sengupta

**Affiliations:** 1 Department of Biomedical, Biological and Chemical Engineering, University of Missouri, Columbia, Missouri, United States of America; 2 ImpeDx Diagnostics, Kansas City, Kansas, United States of America; Jamia Hamdard, INDIA

## Abstract

**Background:**

The spread of multi-drug resistant tuberculosis (MDR-TB) is a leading global public-health challenge. Because not all biological mechanisms of resistance are known, culture-based (phenotypic) drug-susceptibility testing (DST) provides important information that influences clinical decision-making. Current phenotypic tests typically require pre-culture to ensure bacterial loads are at a testable level (taking 2–4 weeks) followed by 10–14 days to confirm growth or lack thereof.

**Methods and findings:**

We present a 2-step method to obtain DST results within 3 days of sample collection. The first involves selectively concentrating live mycobacterial cells present in relatively large volumes of sputum (~2-10mL) using commercially available magnetic-nanoparticles (MNPs) into smaller volumes, thereby bypassing the need for pre-culture. The second involves using microchannel Electrical Impedance Spectroscopy (m-EIS) to monitor multiple aliquots of small volumes (~10μL) of suspension containing mycobacterial cells, MNPs, and candidate-drugs to determine whether cells grow, die, or remain static under the conditions tested. m-EIS yields an estimate for the solution “bulk capacitance” (Cb), a parameter that is proportional to the number of live bacteria in suspension. We are thus able to detect cell death (bactericidal action of the drug) in addition to cell-growth. We demonstrate proof-of-principle using *M*. *bovis BCG* and *M*. *smegmatis* suspended in artificial sputum. Loads of ~ 2000–10,000 CFU of mycobacteria were extracted from ~5mL of artificial sputum during the decontamination process with efficiencies of 84% -100%. Subsequently, suspensions containing ~105 CFU/mL of mycobacteria with 10 mg/mL of MNPs were monitored in the presence of bacteriostatic and bactericidal drugs at concentrations below, at, and above known MIC (Minimum Inhibitory Concentration) values. m-EIS data (ΔCb) showed data consistent with growth, death or stasis as expected and/or recorded using plate counts. Electrical signals of death were visible as early as 3 hours, and growth was seen in < 3 days for all samples, allowing us to perform DST in < 3 days.

**Conclusion:**

We demonstrated “proof of principle” that (a) live mycobacteria can be isolated from sputum using MNPs with high efficiency (almost all the bacteria that survive decontamination) and (b) that the efficacy of candidate drugs on the mycobacteria thus isolated (in suspensions containing MNPs) could be tested in real-time using m-EIS.

## 2. Introduction

### 2.1. Motivation

Tuberculosis (TB) is one of the world’s significant public health challenges. In 2017, 1.6 million people died due to TB, and 10 million new infections were recorded [1]. Since TB is both fatal and contagious, timely diagnosis and treatment is the key to containing the spread of TB.

One of the major challenges for easy and effective treatment is the emergence of drug-resistant strains of Mycobacterium tuberculosis (Mtb), the organism causing TB. There is a set of 4 drugs that are considered “first line” in the treatment of TB: Rifampicin (a.k.a. rifampin) (RIF), Pyrazinamide (PZA), Ethambutol (EMB), and Isoniazid [2, 3] (Sometimes, Streptomycin (STR) is also included in this list [3]). Of these, RIF and INH are the first and second most common drugs typically prescribed, and strains of Mtb showing resistance to them individually are designated RIF-resistant and INH-resistant, respectively.

A strain of Mtb is considered to be multi-drug resistant (MDR) if it is resistant to both [3]. Also, there exist other “second-line” drugs that can treat TB but are not preferred for a variety of reasons ranging from cost to side effects. These include fluoroquinolones as well as other drugs, some of which need to be injected. A strain of Mtb that is resistant not only to INH and RIF, but also to the fluoroquinolones, and at least one of the 3 most common injectable second-line drugs (i.e., amikacin, kanamycin, or capreomycin) is designated as an Extremely Drug Resistant (XDR) strain.

In 2015, the year’s annual report on TB issued by the World Health Organization (WHO) included a supplement devoted to the emergence and spread of drug resistance [4], and in 2017, the WHO issued updates to the previously issued supplement on drug resistance [5]. According to these reports, 4.1% of new and 19% of previously treated TB cases were estimated to have RR (Rifampicin Resistance) or MDR TB. Additionally, 6.2% of MDR-TB cases had additional drug resistance and were considered Extensively Drug-Resistant TB (XDR-TB). Only 54% of MDR-TB patients who had started treatment in 2014 were successfully treated while 16% of patients died and 8% of patients’ treatments failed (21% were lost to follow-up or not evaluated). This translates to about 600,000 new cases of MDR/RR-TB cases emerging in 2016 with 240,000 cases of death due to MDR/RR-TB.

The primary diagnostic method for detecting TB in low resource settings is the Sputum Smear Microscopy. This method involves exposing the collected sputum to stains that adhere to *M*. *tuberculosis* (Mtb) cells and looking under a microscope for the stained cells. Although this method is inexpensive, it has a relatively high limit of detection, around 105 CFU/mL of Mtb [6], and thus has a high rate of false negatives. This is especially true for patients who are asymptomatic or mildly symptomatic whose Mtb loads may be as low as 1000 CFU in 2-5mL of sputum [2]. Another limitation of this method is that it cannot be used for Drug Susceptibility Testing (DST). To both detect Mtb cells and assay for drug resistance, two broad classes of methods are available: genotypic (molecular) and phenotypic (culture based).

### 2.2. Genotypic (molecular) systems

Genotypic systems detect the species/groups of microorganisms in a biological sample by detecting the presence of DNA with a characteristic sequence. Drug resistance is detected based on finding DNA encoding genes/mutations known previously which are responsible for resistance to one or more drugs.

Examples of this system include the Xpert MTB/RIFTM from Cepheid and Line Probe Assays (LPAs) from companies like Hain Biosciences. The XpertTM yields information regarding the presence of Mtb DNA and that of a gene encoding resistance to RIF. LPAs are available from Hain Bioscience in Germany [7, 8] and detect mutations in the *rpoB* gene, which is associated with RIF resistance, as well as mutations in the *katG* gene and the *inhA* promoter region, which are associated with INH resistance. Another assay, called MTBDRslTM, which is also from Hain, identifies certain genetic mutations that make Mtb resistant to fluoroquinolones.

These systems have several advantages that propelled them to being recently adopted in a number of settings, including low-resource environments. Their most important trait is that they are rapid. For instance, the XpertTM is able to provide an answer in 2–6 hours [9], and the LPAs from Hain have a turnaround time of 24–48 hours [8]. Another important trait of these systems is that they are automated and hence have low user error and, if needed, can handle large numbers of samples.

These systems have two major drawbacks. The first is the relatively high cost, which requires significant subsidies to reach users in low-resource environments. While in the US and Western Europe, the Xpert MTB/RIF instrument retails for around $60,000 and individual test cartridges retail for around $65 [10]. The Foundation for Innovative Diagnostics (FIND) coordinates volume discounts from the manufacturer and subsidies from the UN (WHO), governments and various private philanthropies, such as the Gates Foundation, make the instruments available for around $17,000 and cartridges for around $10 each to select users in low-resource environments [11]. Similarly, LPA strips are made available at a price of approximately $10. However, as the WHO notes [7, 8] performing the LPA test requires other laboratory consumables and supplies which may push the cost to between $20 and $30 a test, even after subsidy.

The other main drawback is that the genetic tests provide only a limited picture of the organism’s drug susceptibility profile, especially from the point of effective treatment. For instance, the Xpert detects resistance to rifampicin only, for which the genetic basis is well-understood, and the LPAs detect some, but not all, cases of isoniazid resistance since there are multiple resistance pathways for resistance to INH [12]. Complicating the picture further, a mutation to the katG gene (S315T) that is detected by the LPAs, which is known to result in elevated values of Minimum Inhibitory Concentrations (MIC) to INH, but the degree to which the MIC is elevated varies significantly among isolates [7]. This implies that certain isolates harboring the mutation can still be treated with INH, but others cannot. Moreover, the absence of known mutations does not imply that the drug of choice will be effective. The CDC states in its guidelines to clinicians [13], “Further, not all biological mechanisms of resistance are known. As a result, if no mutations are detected by the molecular assay, resistance cannot be ruled out. **Therefore, it is essential that conventional growth-based drug-susceptibility tests are done and used in conjunction with molecular results**.”

### 2.3. Phenotypic systems

Phenotypic systems are the systems in which Mtb is detected and/or characterized with respect to its drug susceptibility based on direct or indirect observations of cellular growth and/or bacterial metabolism in a chosen media.

The most common matrix tested for the presence of Mtb is sputum. Since there are large numbers of microorganisms of various types present in the sputum of all patients, a decontamination protocol is typically performed before any phenotypic test. The protocol involves the use of a decongestant (such as NALC–N-acetyl-L-cysteine) to make the sputum less viscous and a killing agent (such as sodium hydroxide) that kills all microorganisms except the extremely hardy mycobacteria like Mtb. Once decontaminated, the sample (decontaminated sputum) is added to a growth media and inserted into an Automated Culture-Based Diagnostic System like the MGIT (Mycobacteria Growth Indicator Tube) from Becton Dickinson (BD) or the Trek ESP system form Thermo-Fisher.

Due to low operational costs and rugged performance, automated culture-based diagnostic systems remain the workhorses for multiple applications, including the detection of TB (MGIT and Trek ESP), and subsequent phenotypic DST. These automated culture systems rely on detecting living bacteria by looking for changes brought about by bacterial metabolism to the properties of suspension, such as O2/CO2 levels, pH, electrical conductivity, temperature, etc. Their major drawback is that, because the amounts of metabolites released by the bacteria are low, they have a high “threshold” of detection. In other words, these systems require that the concentrations of the bacteria in the suspension need to rise to around 108 CFU/mL before a positive culture can be identified [14]. This can be time-consuming since bacteria such as Mtb has a long doubling time and may be present in the sputum in low loads, around 1000 CFU in 2-5mL [2]. Thus, it can sometimes take many days (weeks) before the culture is at high enough levels for detection.

Drug Resistance Testing is typically carried out after the Mtb has been detected in the automated blood culture system. Aliquots from the positive MGIT tube are transferred to other tubes, where the media has been supplemented with antibiotics, and observed over 1–2 weeks to detect growth (or lack thereof) in the presence of antibiotics. This, coupled with the fact that the DST starts many days after the sputum sample is collected, results in the time to results for the phenotypic DST being ~ 1 month, or longer.

To overcome the need for “pre-culture” prior to DST, a method called Microscopic Observation of Drug Susceptibility (MODS) has been developed [15]. The test uses 24- well plates with four wells for a single patient specimen: two wells are drug-free, while the other two wells contain Rifampicin [16] and Isoniazid [2]. After inoculation, the plates are sealed in zip-lock bags and then incubated. If the pathogen in sputum is present, their growth in the liquid medium is observed under an inverted light microscope and morphologically examined for patterns specific to Mtb. This typically takes 7–14 days to observe growth in the wells without drugs and to confirm lack of growth in those with drugs [17] and the cost per test was estimated to be about $5 in a low-resource environment of Peru [18]. Microscopic Observation Drug Susceptibility assay (MODS) has hence been recommended by the WHO as an affordable method for relatively rapid detection and DST.

Despite its advantages, MODS is not as widely used in resource-limited settings as was initially anticipated due to concerns for biosafety, reliance on the skill of laboratory personnel in interpreting results, and efficiency for handling a large number of samples. While concerns about biosafety can be alleviated using commercially produced kits with sealed wells, such as those produced by hardy Diagnostics [18], and a certain degree of automation can be introduced in the capture of images [19], this method still requires that every well be visually inspected by a well-trained laboratory professional. This puts a requirement of both time and human resources that often make it unfeasible to handle a large number of sputum samples that may need to be tested in high-TB burdened and low-resource settings.

The phenotypic approach presented here seeks to (a) overcome the need for pre-culture by using a magnetic-nanoparticles (MNP) based method to collect virtually all the mycobacteria cells present in a relatively large volume of sputum (2–5 mL) into a relatively small volume (~200μL) of buffer/growth the medium within a short period of time (~20 minutes) (b) overcome biosafety concerns by using microfluidic technology to partition the 200μL of media containing mycobacteria cells into sixteen individually isolated micro-reservoir (wells) of 10μL volume, each containing different amounts of candidate drugs, and (c) overcome the need for subjective judgment by a trained clinical worker by using an extremely sensitive electrical method (m-EIS) that monitors the suspensions in the wells continuously, and is able to report not only the growth but also the death of cells in real-time.

**Fig 1:**
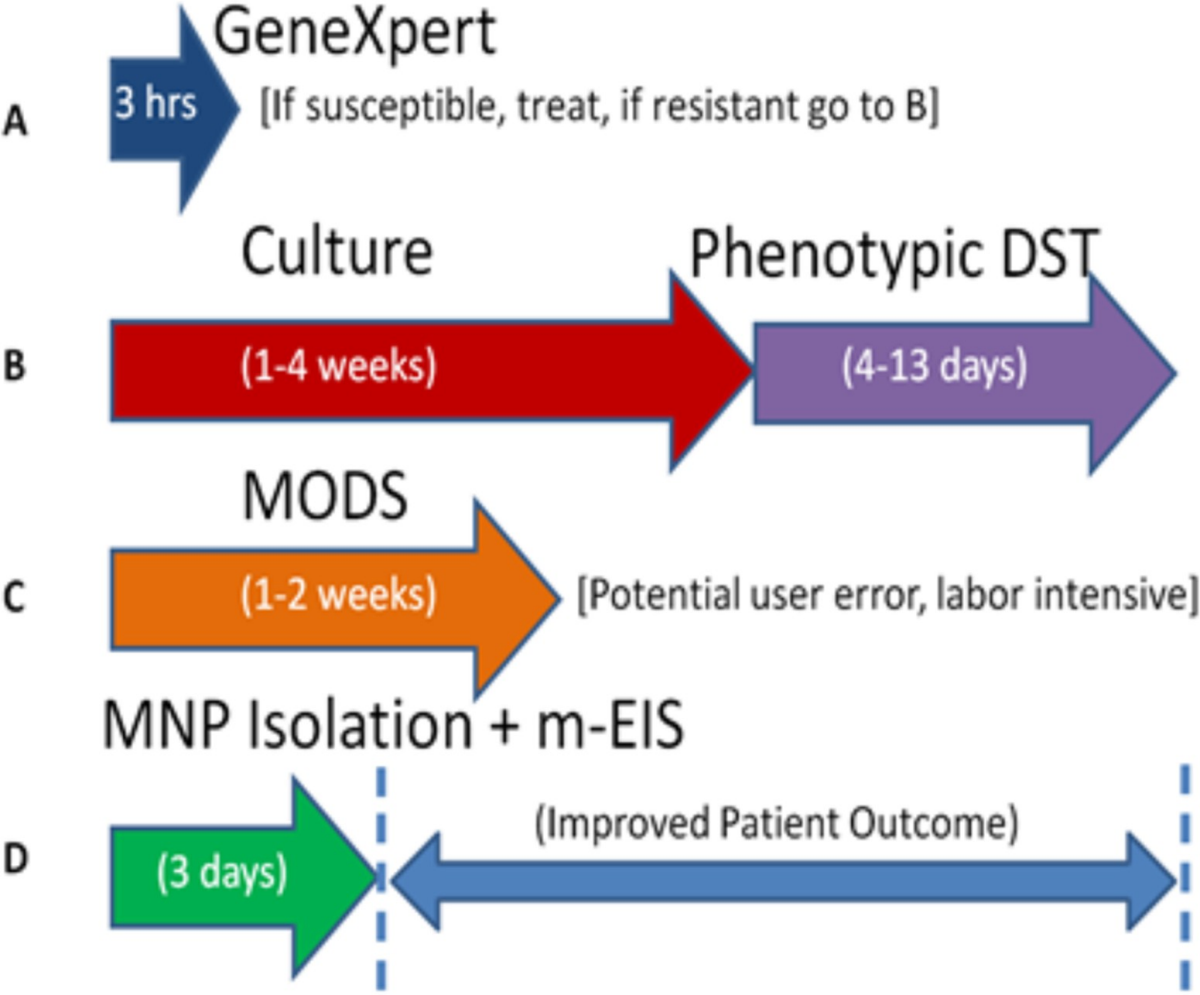
Timeline of available approaches to obtaining drug susceptibility information for TB. (A) GeneXpert genotypic approach that only identifies Rifampicin susceptible pathogens. If RIF-resistant, the sample is channeled to path B. (B) Traditional culture based phenotypic approach (MGIT etc.) (C) Microscopic Observation of Drug Susceptibility (MODS). (D) Proposed approach (isolation using MNPs + growth/death/ stasis assay using EIS).

Because this method pre-concentrates the mycobacteria prior to culture, and is able to monitor cell death in real-time due to the action of antibiotics, it potentially enables the phenotypic susceptibility tests to be performed at time-scale comparable to the “molecular” methods, and with the automation and scalability of the latter, but at a fraction of the cost. Fig 1 shows an outline for the timelines of each method.

### 2.4. m-EIS

Our lab has previously developed and patented [20, 21] a novel method to monitor the proliferation or death of microorganisms in suspension, which we refer to as microchannel Electrical Impedance Spectroscopy (m-EIS). It relies on the fact that, in the presence of high-frequency alternating current (AC) electric field, the membranes of cells become polarized and store charge; thereby acting like electrical capacitors [22]. These capacitances at the individual cells contribute to the overall “bulk capacitance” of the suspension or net charge stored in the interior. The amount of charge stored by a bacterium is about 100X that of an equal volume of aqueous solution [23]. Therefore, even at low concentrations (volume fractions), bacteria in a suspension contribute significantly to the latter’s bulk capacitance. Also, it should be mentioned that only living cells with intact membranes contribute to bulk capacitance. A cells death is accompanied by a loss of membrane potential and electrical polarization [24].

Measuring the bulk capacitance is not straight-forward since the electrical behavior of aqueous solutions containing polarizable species, like cells and proteins, is extremely complex. Such systems can be modeled electrically by the circuit shown in Fig 3A [25]. In this equation, the charge stored at the electrode-solution interfaces is accounted for by the two electrode interface capacitances (Ce) and charge stored by elements dispersed in the interior, by “bulk capacitance” (Cb). Measuring changes in Cb is challenging since Ce is roughly 1000 times larger. The key innovation [26] is a method that enables the changes in Cb to be detected, despite the “screening effect” of charges at the electrode interface by making the microfluidic channels long and narrow, with the electrodes placed along the length of the channel, which increases the effective bulk resistance (Rb) of the suspension. This increases the impedance of the bulk (RbCb) to be comparable to that of the interface at realizable frequencies allowing an appreciable voltage drop over the bulk-suspension. Therefore, the charge-storage in the bacteria contributes significantly to the measured impedance. The long narrow channel causes more of the electrical “lines of force” to interact with the suspended bacteria. The measured impedance (Z) at 500 frequencies (ω) between 1 kHz and 100 MHz is fit to the equation, and Cb is estimated (along with Rb, Re and Ce). The application of this technology has been demonstrated for food quality assessment [26] and blood culture [27]. For blood culture, the “times to detection” (TTDs) were often days shorter than the current market leader. The threshold concentrations of detection are 103–104 CFU/mL, while compared to current technologies 108 CFU/ml [14].

Further, since single cells lose their membrane potential when they die and no charge accumulates at the membrane in the absence of a potential, m-EIS is able to observe cell death in real-time. This insight has been previously used to determine minimum inhibitory concentrations (MICs) for multiple bacteria-antibiotic pairs [28]. Besides being rapid, taking 4 hours for *E*. *coli*, *S*. *aureus* and *Pseudomonas* detection, and accurate, correct MIC values were obtained for well-characterized stains; it is able to distinguish between bacteriostatic effects, in which the bacteria can no longer replicate, from bactericidal, which will kill the bacteria cell. Moreover, cell death can be observed using m-EIS for initial loads at or above the “threshold” concentrations of around 103 CFU/mL. This was shown for *E*. *coli* and *Pseudomonas aeruginosa* [29]. Not only does the m-EIS method require a lower threshold concentration, but it also works with small volumes of the sample (~10μL). It thus requires a very small number of cells (< 100 CFUs) to work. Further (as will be demonstrated in this work), it is unaffected by the presence of inert materials like MNPs, even if they happen to be charged or polarizable. The presence of inert species merely contributes to the background, and since m-EIS looks for a *change i*n bulk capacitance from the baseline (time t = 0) value brought about either by cell proliferation or cell death to determine the effect of the candidate drug on the mycobacterial cells, they do not affect the interpretation of the data as long as their number remains constant. Due to a combination of needing low cell numbers to work, and an ability to work in the presence of MNPs, m-EIS is ideally suited to monitor cell growth/death after the cells present in the entire sputum sample have been concentrated down into a small volume using MNPs.

## 3. Materials and methods

### 3.1. Rationale and overview

The goal of developing this method is to obtain rapid and accurate phenotypic DST results. The main barriers to these results are (a) the relatively low load of mycobacteria that can be present in sputum, which leads to the requirement that it be pre-cultured and (b) the relatively slow metabolic rate (doubling time) of the mycobacteria, which makes it time-consuming to distinguish a suspension containing cells that grow despite the presence of drugs, from those in which cells cease to grow, or die. We overcome the former by isolating and concentrating cells into a small volume using MNPs, and the latter by using a sensitive electrical method that is able to monitor cell growth/death/stasis in small volumes in real-time.

Since *M*. *tuberculosis* is a Biosafety Level 3 (BSL-3) organism, and our lab is only certified for BSL-2 ones, we chose to use BSL-1 and BSL-2 analogs. *M*. *smegmatis* is a BSL-1 organism, whose membrane is very similar to that of *M*. *tuberculosis* [30, 31], and *M*. *bovis BCG* is a BSL-2 organism that has a doubling time of ~20 hours [32], which is comparable to the ~24 hour doubling time of *M*. *tuberculosis* [30, 31]. To simulate a patient sample from clinical labs, artificial sputum was created in lieu of actual patient sputum. Since naturally-occurring sputum also contains non-mycobacterial microorganisms (Gram-positive and Gram-negative bacteria), we also added to our sputum *S*. *aureus* and *P*. *aeruginosa* to represent the effects of the presence of other commensal/ pathogenic bacteria in the sputum.

**Fig 2:**
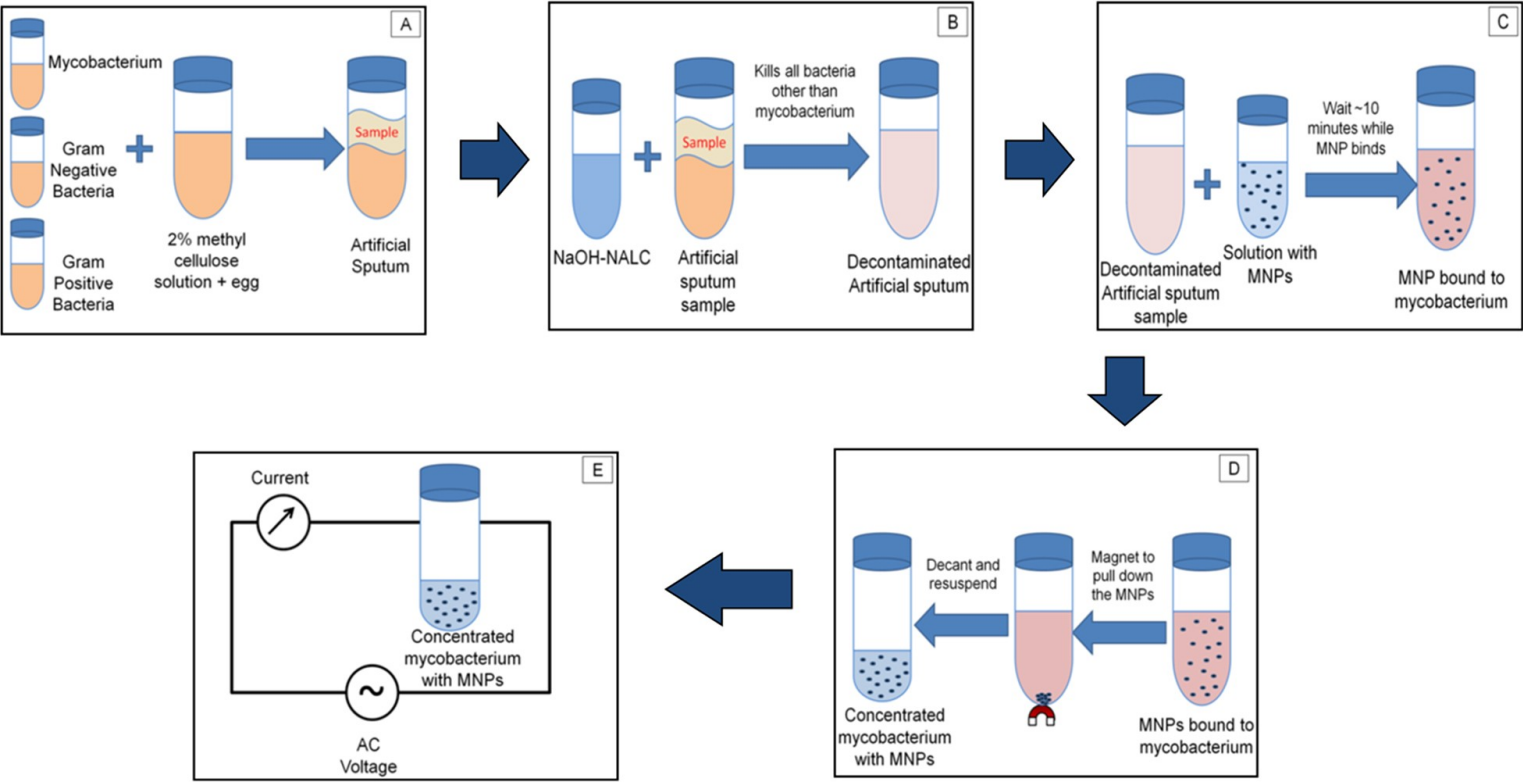
Experimental protocol. (A) the preparation of artificial sputum with mycobacteria + Gram-positive and Gram-negative bacteria (B) the digestion/decontamination (C) Addition of MNP and allowing them to bind to the mycobacteria (D) Isolation of the mycobacterium into a pellet and its resuspension (E) Periodic electrical assay using m-EIS.

An overview of the experimental protocol followed is depicted in Fig 2. Once synthetic “infected” sputum is created with Gram-positive, Gram-negative, and mycobacteria, it is subjected to a decongestion and decontamination protocol similar to one that a patient sample would undergo. After decongestion and decontamination, MNPs are used to collect mycobacteria. The efficiency of the decontamination process and the collection efficiency for mycobacteria surviving decontamination are evaluated using plate counts. Mycobacterial suspensions containing loads of microorganisms and MNPs similar to those obtained at the end of the isolation process are then exposed to candidate antibiotics at known concentration and the efficacy of the m-EIS method to observe cell proliferation/death in real-time are then evaluated. To establish the fact that the m-EIS measurements can be conducted despite the presence of MNPs, we also conduct similar tests without the presence of MNPs. More detailed information containing the individual steps is provided below (including data collection, analysis, and statistical analysis).

### 3.2. Bacterial preparation

All cultures used during the experiment were purchased through ATCC and subcultured in the lab. For the in-vitro experiments, two different types of mycobacteria were chosen as surrogates in lieu of *M*. *tuberculosis*, viz. *Mycobacterium smegmatis* (ATCC 700084) and *Mycobacterium bovis BCG* (ATCC 3574). To simulate the bacteria found naturally in sputum from patient samples, *Staphylococcus aureus (ATCC 29213)* and *Pseudomonas aeruginosa (ATCC 27853)* were selected as representative Gram-positive and Gram-negative bacteria, respectively. *M*. *smegmatis* was cultured in Middlebrook 7H9 (Fluka Analytical, St. Louis Missouri) and *M*. *bovis BCG* were cultured in Middlebrook Albumin Dextrose Catalase (ADC) supplements (HIMEDIA, Mumbai, India). Both *S*. *aureus* and *P*. *aeruginosa* were cultured in tryptic soy broth (TSB) media (*Sigma Aldrich*, *St*. *Louis*, *Missouri)*. All cultures were incubated at 37°C. To obtain the proper concentrations for the experiments, a UV-VIS spectrophotometer (Azzota S M 1000) was used to find the optical density (OD) of the samples at 600nm or 570nm. From literature, it is known that OD600 values of 0.1 and 0.05 correspond to concentrations of 1 x 107 CFU/mL for *M*. *smegmatis* and *M*. *bovis BCG*, respectively [33, 34]. Similarly, the OD570 value for 1 x 107 CFU/mL of *S*. *aureus* is reported to be 0.15 [35], and that of ~1x 108 CFU/mL *P*. *aeruginosa* is reported to be 0.1 [36]. All bacteria were grown to the above-mentioned target concentrations, and then serially-diluted to obtain concentrations desirable for introduction into the artificial sputum matrix.

### 3.3. Artificial sputum preparations

To accurately represent the patient sample, we created artificial sputum for the extraction experiments. The artificial sputum was prepared according to protocols available in the literature [37–39]. This sample was a 1% (w/v) aqueous methylcellulose solution which had a starting volume of 1L.This sample was then sterilized using an autoclave and 1 emulsified egg was added to the mixture to be used as artificial sputum for our experiments. To properly simulate bacterial collection from sputum to bacterial concentrations of *S*. *aureus* and *P*. *aeruginosa* was 10,000 CFU/mL. The *M*. *smegmatis* and *M*. *bovis BCG* concentrations were 10,000 CFU/mL. For the experiment, 5mL of sputum and bacteria were used to simulate the sample. Further representation of this step can be seen in Fig 2A.

### 3.4. Decontamination and digestion

This process is conducted to ensure that (a) all non-mycobacterial species are eradicated from the collected specimen and (b) living mycobacteria are released from the matrix (aqueous buffer) and can grow freely in suspension. The most commonly used digestion/decontamination reagent consists of a mixture of N-acetyl-L-cysteine (NALC) and Sodium Hydroxide, where NALC acts as the digestion agent and the NaOH acts as the decontaminating agent.

For the experiments presented here, the standard NaOH-NALC decontamination process suggested in literature [40] was used with minor modifications, viz. we used a slightly lower concentration of NaOH of 1%, vs. the 2% commonly used to [2], along with a shorter duration of exposure (10 minutes for *M*. *smegmatis* and for *M*. *bovis BCG* vs 15 minutes for *M*. *tuberculosis*). This was due to the fact that *M*. *smegmatis* and *M*. *bovis BCG* are less hardy than *M*. *tuberculosis*, making them more susceptible to the decontamination process.

The efficacy of the process (both in terms of eliminating non-mycobacterial species, and in allowing the mycobacteria to survive) was evaluated by plating aliquots obtained at the beginning and the end of the decontamination and extraction process. At the beginning of the process, 5mL of the artificial sputum containing *S*. *aureus*, *P*. *aeruginosa*, and *M*. *smegmatis* / *M*. *bovis BCG* at ~ 104 CFU/mL was prepared. To verify this, 100μL aliquots of the “infected” artificial sputum were plated on Tryptic Soy Agar (TSA) and 7H10 plates (for samples containing *M*. *smegmatis* and other non-mycobacterial species), or TSA and Lowenstein-Jensen (LJ) plates (for samples containing *M*. *bovis BCG* and other non-mycobacterial species) after appropriate dilutions. Colony counts from the TSA plates indicate the numbers of non-mycobacterial species present, and those on 7H10/LJ plates indicate the numbers present for mycobacteria.

To the artificially prepared “infected sputum”, an equal volume (5mL) of 2% NaLC-NaOH was added, mixed well and allowed to sit for 10 minutes. At the end of 10 minutes, the sample was neutralized by adding 450μL of ~ 12M HCl. Once again 100μL aliquots from the suspension were plated on TSA, 7H10 and/or LJ agar plates to estimate the numbers of mycobacteria and non-mycobacterial species surviving.

### 3.5. Extraction using MNPs

The MNPs used for the experiments reported here were commercially available RapiPrep-TBTM beads (Microsens Biotechnologies, London UK) that have already been optimized to bind to *M*. *tuberculosis* cells present in sputum [41–43].

These beads are supplied in a suspension wherein the concentration of beads is 2.5 mg/mL. 4mL of this suspension is added to the ~10mL of neutralized decontaminated sputum, gently mixed by inverting by hand for ~10 times, and allowed to sit for 10 minutes. After this duration, it was mixed again by inversion, and the tube placed against a permanent magnet for 1 minute. This draws virtually all the MNPs to the side of the tube, forming a pellet, which also contains mycobacteria adhered to the MNPs. The supernatant is discarded, and the pellet is re-suspended in 1mL of the buffer. 100μL aliquots of this resuspension are plated on TSA/ 7H10/ LJ plates to estimate the numbers of live cells collected. It may be noted that if virtually all the MNPs are collected into the pellet, the re-suspension would have an MNP concentration of ~10 mg/mL, which is the concentration used during the m-EIS studies.

**Fig 3:**
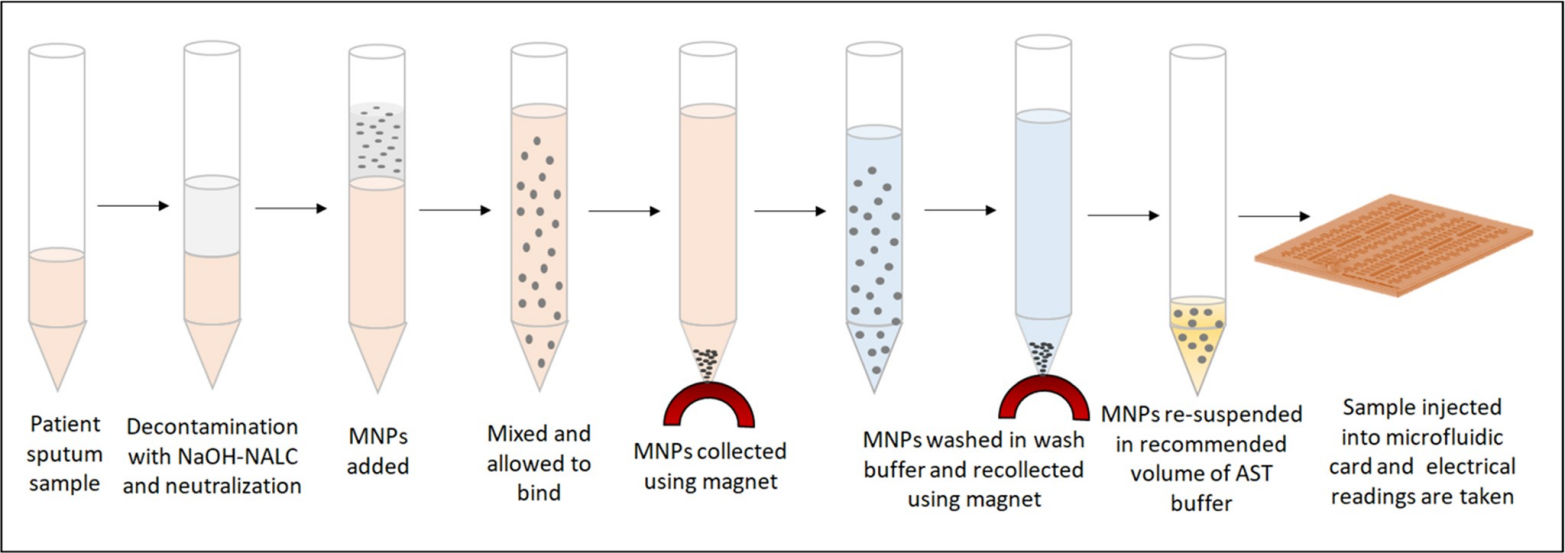
Real-world clinical patient sputum sample processing protocol. Schematic of proposed approach involving isolation of mycobacteria using MNPs to bypass the preculture, allowing the assay of growth/death using m-EIS measurements to be completed quickly.

The real-world clinical patient sputum samples (which contain mycobacteria along with other commensal microorganisms) can be processed similarly for decontamination and extraction using magnetic nanoparticles. An overview of the patient sputum sample processing protocol prior to use in m-EIS for antibiotic susceptibility testing can be seen in Fig 3.

### 3.6. Choice of drugs and concentrations tested

The drugs tested in this study were a subset of common/first-line drugs typically used against *M*. *tuberculosis* and whose effects on *M*. *smegmatis* and/or *M*. *bovis BCG* at various concentrations have been studied previously by others. As shown in Table 1, drugs were chosen to represent the various effects that the drugs can have with respect to microbial survival: viz. cidal (where the drug kills the microorganism), static (where the net number of live microorganisms does not change), and ineffective (where the microorganism keeps proliferating despite the presence of drug). For each cidal/static drug, three concentrations were chosen: one significantly below the known Minimum Inhibitory Concentration (MIC) range, one within the known MIC range, and one much above the same.

**Table 1:** Specific concentrations of antibiotic tested for *M. smegmatis* and *M. bovis BCG*.

		Concentration Tested (μg/mL)		
Organism	Drug (type)	< MIC	MIC	> MIC
*Mycobacterium smegmatis*	Amikacin (cidal)	0.0625	1	16
	Ethambutol (static)	0.125	4	32
	Rifampicin (ineffective)	[upper limit of range typically tested] 16		
*Mycobacterium bovis BCG*	Amikacin (cidal)	0.125	1	16
	Ethambutol (static)	0.125	4	32
	Pyrazinamide (ineffective)	[upper limit of range typically tested] 16		

For *M*. *smegmatis*, the drugs selected are: amikacin (batericidal, with a known MIC of 0.5 mg/L [44]), rifampicin (known to be ineffective [45]), and ethambutol (known to be bacteriostatic, with an MIC of 1 mg/L [44]). For *M*. *bovis BCG*, the drugs selected are: amikacin (bactericidal, with a known MIC of 0.125 mg/L [46], pyrazinamide (known to be ineffective [47]), and ethambutol (known to be bacteriostatic, with an MIC range of 2mg/L to 4 mg/L) [48]).

In a real-world situation, we anticipate that the mycobacteria isolated from human sputum using MNPs will be dispersed into growth media and tested against multiple candidate drugs in parallel. We hence prepared suspensions with mycobacteria and MNPs in growth media where both the initial load of mycobacteria (~105 CFU/mL) and the concentration of MNPs present (10 mg/mL), were similar to what one would expect to obtain at the end of the decontamination and isolation process described above. Since it was uncertain / un-verified a-priori if (a) the MNPs would affect the metabolism of cells in a manner that alters their response to the drugs, and (b) the MNPs would adversely affect the electrical measurements, parallel experiments, where MNPs were not added, were also conducted to serve as a baseline when trying to evaluate the effect of the MNPs. For any given experiment, ~20mL of suspension (containing mycobacteria, growth media, candidate drugs at selected concentrations, and/or MNPs) was prepared and incubated at 37C. Periodically, 50μL aliquots were taken from these liquid cultures and subjected to electrical readings.

### 3.7. Electrical readings

**Fig 4:**
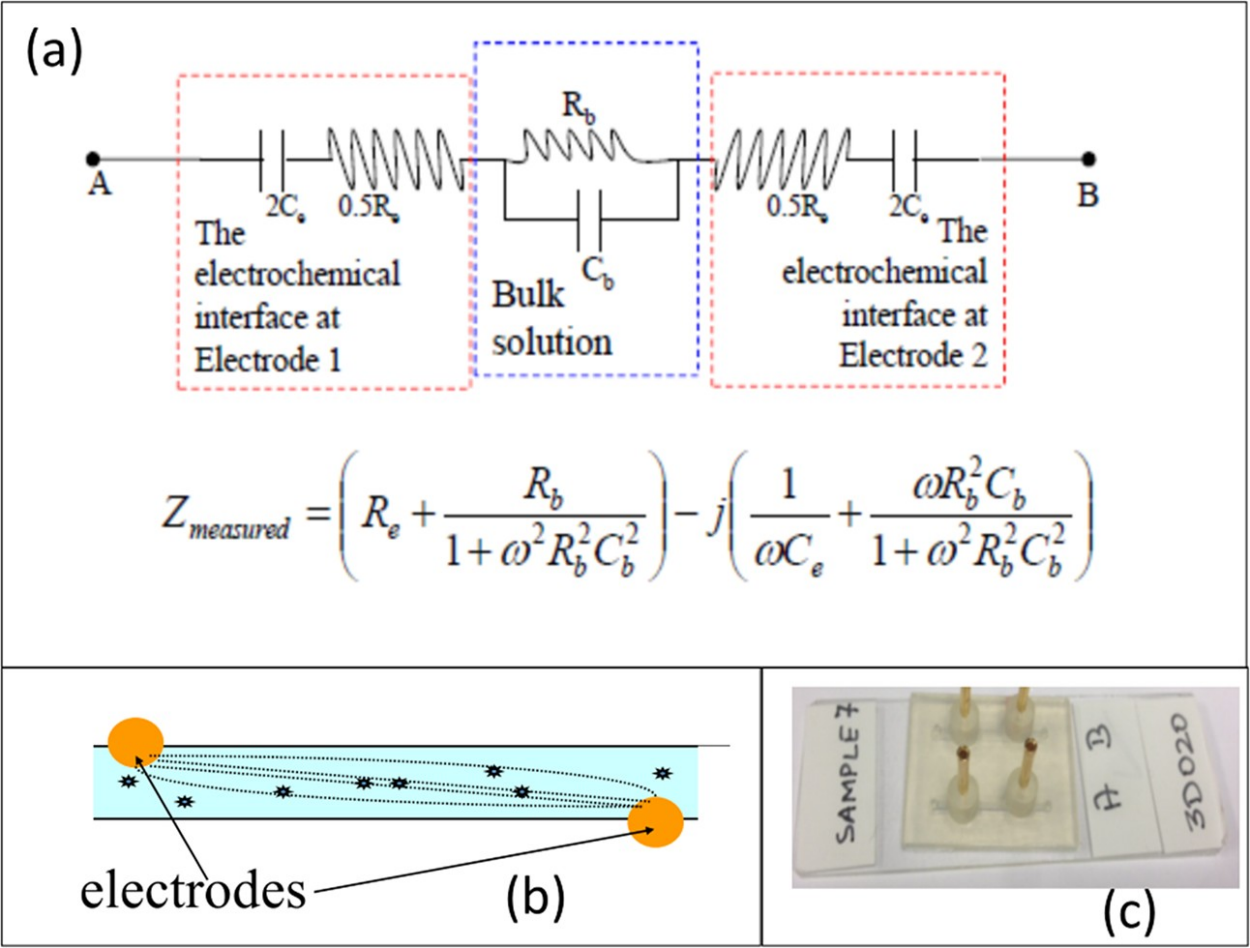
Schematic and electrical circuit model representation of microchannel. (a) Electrical Model of an aqueous suspension in contact with metal electrodes. The equation relates the real (in-phase) and imaginary (out-of-phase) components of the measured impedance (Z) and how they vary as a function of frequency and model parameters (Re, Ce, Rb and Cb) (b) schematic, and (c) picture of microfluidic cassette.

Aliquots (~50μL) were taken from the liquid culture and introduced into a microfluidic cassette containing channels with a 1mm x 1mm cross-section. These cassettes consist of a glass-slide base and a 3D printed top with engraved channels affixed using an adhesive (Epo-Tek 301TM). The 3D printed part was fabricated at the Mizzou 3D Printing Lab and had electrodes asymmetrically placed 10mm apart, as shown in Fig 4. It thus allows the user to assay a fluid volume of ~10μL (~10mm x 1mm x 1mm).

The electrodes shown were connected to an impedance analyzer (Agilent/Keysight 4294A), and impedance measurements [Resistance (R) and Reactance (X)] were recorded at 128 frequencies (ω) between 1 kHz and 100 MHz. This raw data [R(ω) and X(ω)] was analyzed offline using ZViewTM software to yield an estimate of the suspension’s bulk capacitance (Cb). To study the effect of candidate drugs on the mycobacterial cells, such scans were conducted on the same sample multiple times over the duration of the study. The time interval between the readings was ½ to ¾ hour for *M*. *smegmatis* and 12 hours for *M*. *bovis BCG* (except for the case of studying the effect of the cidal drug amikacin on *M*. *bovis BCG*, where readings were taken every hour). At each time-point, 5 independent aliquots were taken from the liquid culture suspension. After loading each aliquot (~50 μL) into the cassette, electrical measurements were taken. Then the channel was cleared twice using sterile base media (7H9 for *M*. *smegmatis*, MGIT for *M*. *bovis BCG*). After the channel was cleared, the channel was loaded again with the next ~50μL aliquot until 5 readings were completed.

While different suspensions had differing initial values of Cb (presumably due to minor differences in initial load of live microorganisms and/or MNPs), we are interested in tracking changes to Cb due to cell growth/death. Hence, all readings were scaled with respect to the average value of Cb at time t = 0.

### 3.8. Analysis of the electrical data

**Fig 5:**
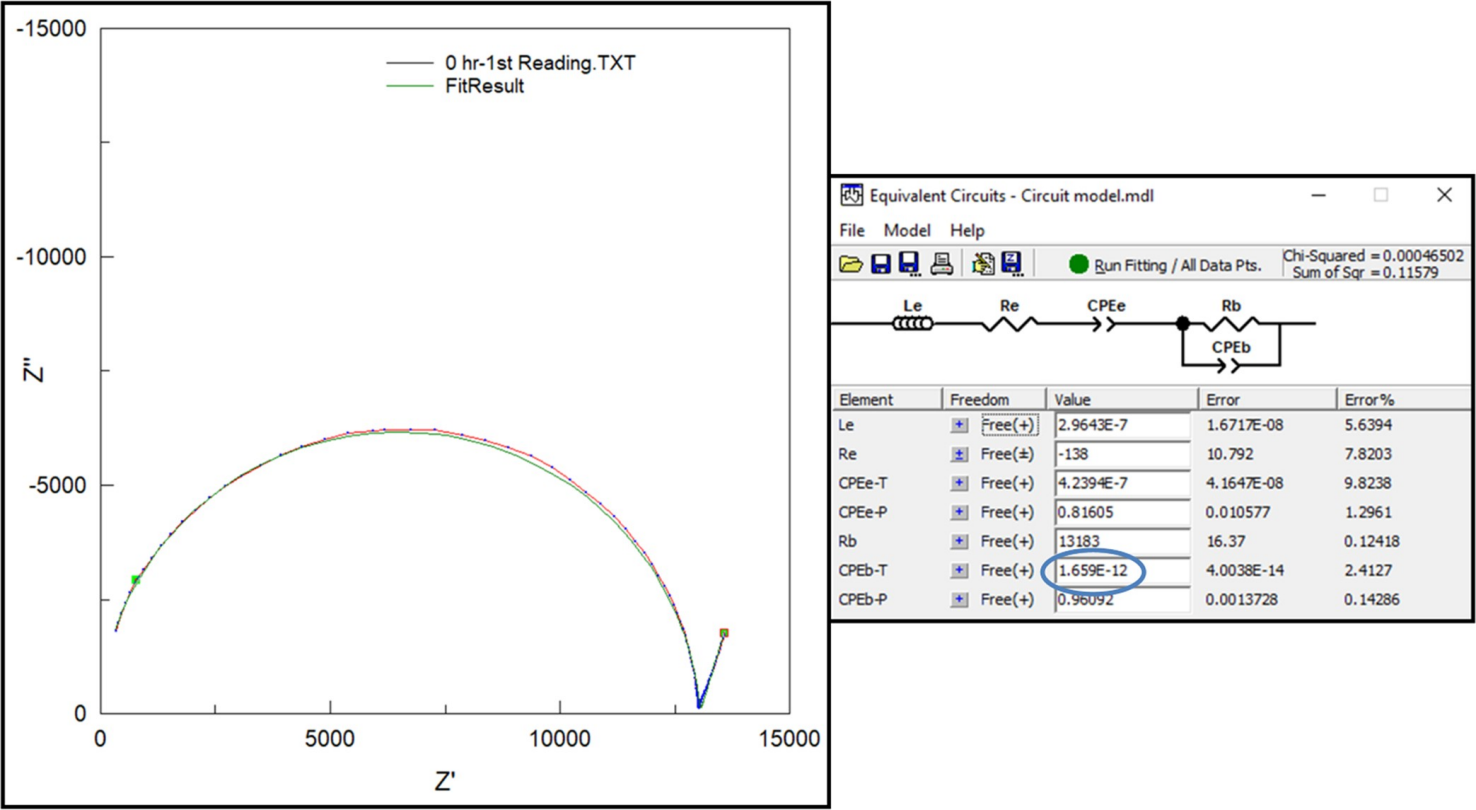
Data analysis using the program ZView®. The left side shows the graphical fit-line to the Resistance (Z’) and Reactance (Z”) which is fit to the circuit of choice (top right). The fit results in an estimate of the bulk capacitance (circled, bottom right).

The data collected by the impedance analyzer is in the form of resistance (R) and reactance (X) values of the bacterial suspension at 128 logarithmically equi-spaced frequencies (w) between 1 KHz and 100 MHz. As described by us previously [49] the R and X vs. ω data is fitted to the equivalent electrical circuit shown in Fig 5 using a commercially available software package (ZView™). The software accepts as an input the Z vs. ω data and provides an estimate for all the circuit parameters, including the “bulk capacitance” (our parameter of interest that provides a measure of charges stored in the interior of the suspension (away from the electrodes)). It may be noted that here, (a) the capacitances and resistances at both electrodes are combined into a single capacitance and resistance, respectively, and (b) both the bulk and interfacial capacitances are represented as a constant phase element (CPE) to account for the non-ideal nature of the capacitance at cell membranes, where charges carried by ions take a finite time to accumulate, unlike an ideal capacitor where the accumulation is instantaneous. The magnitude of the bulk CPE, thus, reflects the amount of charge stored at the membranes of living microorganisms in suspension. An increase in the number of living microorganisms (brought about by cell proliferation) should be reflected as an increase in this value (magnitude of CPE). On the other hand, a decrease in the number of living microorganisms in the suspension (due to the action of the drug present) should lead to a lower bulk capacitance (CPEb − T).

When trying to evaluate the efficacy of a candidate drug (whether the mycobacterium is able to grow in the presence of the drug, is inhibited from growing, or is killed), our problem reduces to establishing whether one can ascertain in an unbiased manner if the value of the bulk capacitances increases, decreases or remains unchanged over the observed duration. To do so, we fit a linear equation to the data of (average) Cb vs. time using Microsoft Excel, and examine the slope. Excel also provides a 95% the confidence interval for the calculated slope. The upper and lower bounds of this confidence interval are also recorded to interpret whether the Cb increases, decreases, or remains unchanged. If the confidence interval encompasses zero (i.e. the upper bound of this confidence interval is positive, whereas the lower bound is negative), then one cannot assert with confidence that the Cb is either increasing or decreasing, and the sample is deemed to be static. On the other hand if both upper and lower bounds are positive, then one can assert that the Cb is increasing (and hence the microorganisms are proliferating) and if both the upper and lower bounds are negative, then one can assert that the Cb is decreasing, presumably because the microorganisms are being killed by the drug.

## 4. Results

### 4.1. Sample preparation (decontamination + extraction)

**Fig 6:**
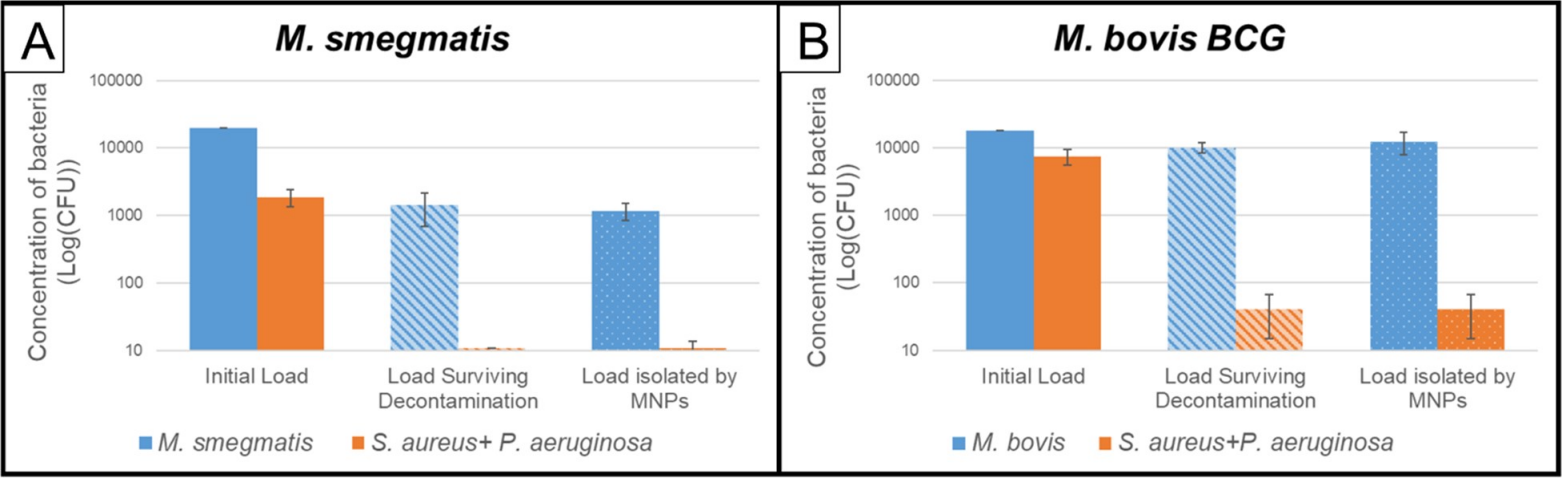
Decontamination and extraction results. Data showing decontamination and extraction of viable mycobacteria from synthetic sputum using RapiPREP-TB TM beads for (A) *M. smegmatis* (n = 3) and (B) *M. bovis BCG* (n = 2) (>95% of cells viable after de-contamination is collected to the pellet).

As discussed earlier and illustrated in Fig 1, our method requires that most mycobacterium present in a patient sample (sputum) survive the decontamination process and be subsequently collected by the MNPs into a “pellet” for resuspension into a small volume of DST media. The protocols adopted for decontamination and MNP-based isolation were also described earlier, along with the protocols adopted to evaluate the number of living bacteria of various types present in the sample of interest. Results (n = 3 for *M*. *smegmatis*, n = 2 for *M*. *bovis BCG*) are presented in Table 2 and Fig 6.

**Table 2:** Loads of viable mycobacteria and “contaminant” bacteria present at each stage of the isolation process.

Type	Sample	Initial Load (CFU)	Load Surviving Decontamination (CFU)	Load isolated by MNPs (CFU)
***M*. *smegmatis***	Mycobacteria	2,000 ± 0	1,413 ± 730	1,180 ± 326
	Contaminant	1,873 ± 532	11 ± 0	11 ± 2.82
***M*. *bovis BCG***	Mycobacteria	18,000 ± 0	10,200 ± 1800	12,500 ± 4500
	Contaminant	7,500 ± 1975	41 ± 26	41 ± 26

### 4.2. m-EIS assay of mycobacterial growth/death

**Fig 7:**
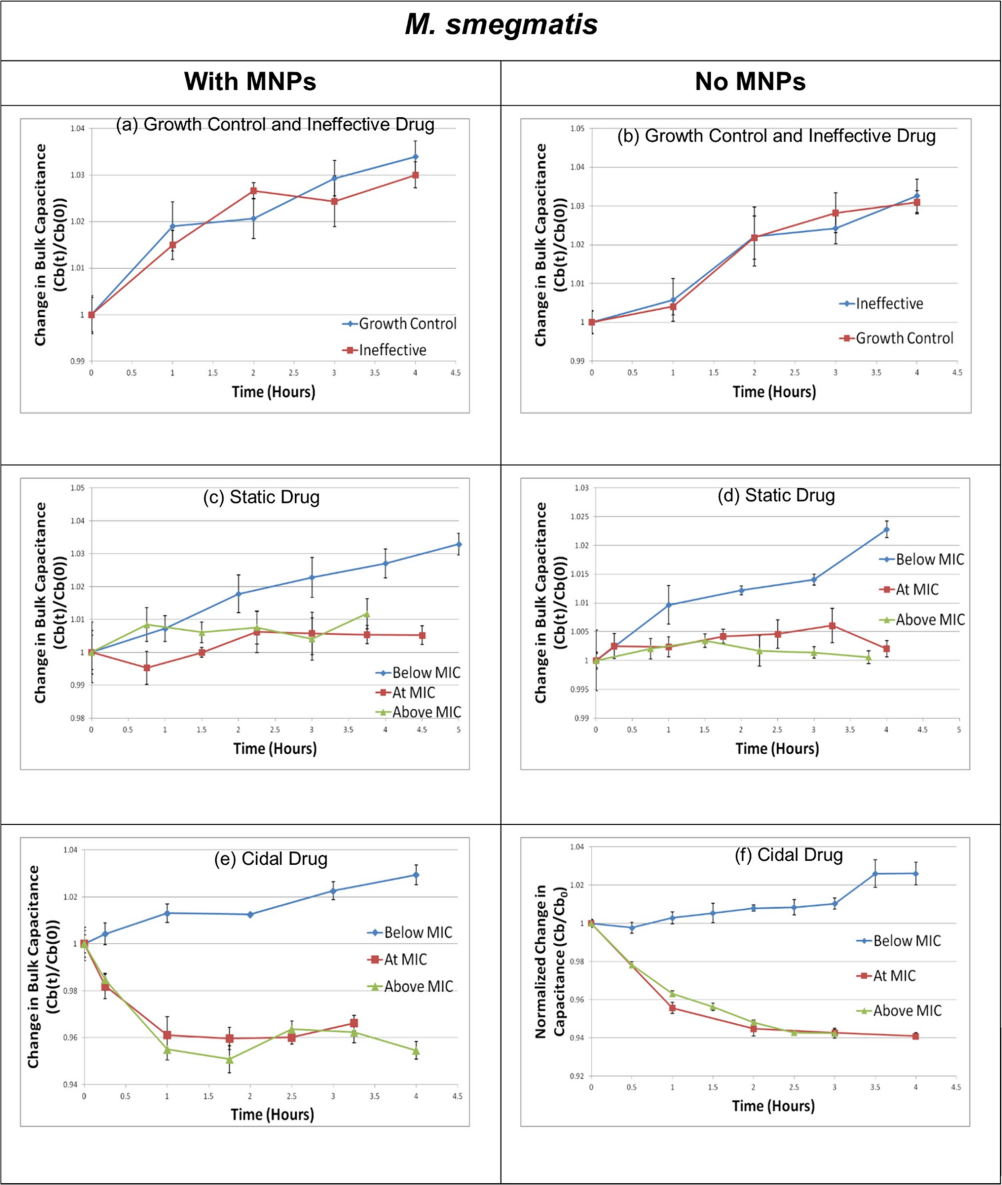
Bulk capacitance changes over time for *M. smegmatis*. Change in the value of the measured bulk capacitance (Cb) as a function of time for *M. smegmatis* cultures with and without Magnetic Nano-particles (MNPs) when exposed to cidal, static, and ineffective antibiotics. All changes are normalized to the baseline (time t = 0) value. (n = 5 at each data point).

**Fig 8:**
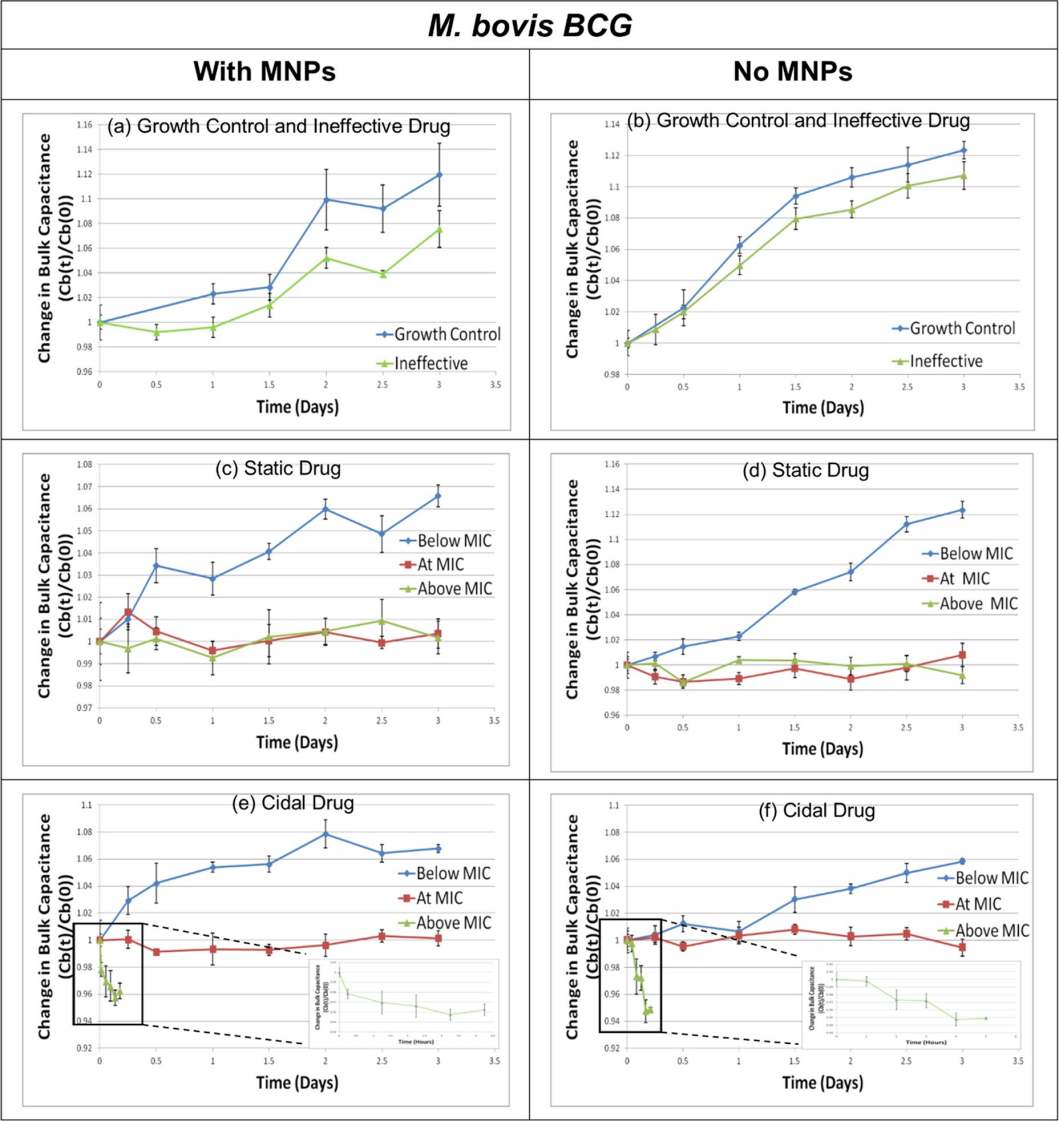
Bulk capacitance changes over time for *M. bovis BCG*. Change in the value of the measured bulk capacitance (Cb) as a function of time for *M. bovis BCG* cultures with and without Magnetic Nano-particles (MNPs) when exposed to cidal, static, and ineffective antibiotics. All changes are normalized to the baseline (time t = 0) value. (n = 5 at each data point).

As explained earlier, m-EIS experiments were conducted wherein candidate drugs at different concentrations were allowed to act on samples mimicking those that we expect to obtain after the sample-preparation process (in terms of loads of living mycobacteria and the MNPs present), and the measured bulk capacitance tracked over a period of time (3 to 5 hours for *M*. *smegmatis* and up to 3 days for *M*. *bovis BCG*). To verify that the MNPs do not affect either the behavior of the mycobacterial cells (with respect to their susceptibility to the drugs tested), or the m-EIS method to monitor the said growth or death, other experiments are run in parallel where conditions are similar to the first set of experiments, but where MNPs are not present. Results (values of bulk capacitance, scaled to the time t = 0 “baseline” value) are shown in Figs 7 and 8 for *M*. *smegmatis*, and *M*. *bovis BCG* respectively. Tables 3 and 4 shows the inference one can draw regarding growth/death/stasis of the mycobacteria based on how these measured values of bulk capacitance change over time. The tables show the estimates of the slope of the straight line fitted to the average of the measured bulk capacitance vs. time data with a 95% confidence interval (CI). If upper and the lower bounds of the confidence interval lies entirely in the positive range, mycobacteria in the sample are deemed to be proliferating; if the CI is entirely within the negative range, mycobacteria are deemed to be dying, and if the CI overlaps zero, mycobacteria are deemed to be static under the conditions tested.

**Table 3:** Estimates of slope of the straight line fitted to data consisting of measured bulk capacitance value (Cb) (dependent variable) as a function of time (t) (independent variable) for various conditions, along with the lower and upper bounds of the 95% confidence interval for *M. smegmatis*.

*M*. *smegmatis*						
Sample Type	Antibiotic	Concentration of Antibiotic (μg/mL)	Estimate of Slope	95% CI Lower Bound	95% CI Upper Bound	Inference
**With MNP**	Growth Control	0	0.0078	0.0030	0.0127	Growth
	Rifampicin	16	0.0069	0.0010	0.0129	Growth
	Ethambutol	0.125	0.0065	0.0051	0.0079	Growth
		4	0.0014	-0.0002	0.0030	Stasis
		32	0.0018	-0.0013	0.0049	Stasis
	Amikacin	0.0625	0.0068	0.0045	0.0090	Growth
		1	-0.0091	-0.0215	0.0033	Stasis
		16	-0.0082	-0.0184	0.0019	Stasis
**Without MNP**	Growth Control	0	0.0086	0.0041	0.0131	Growth
	Rifampicin	16	0.0084	0.0048	0.0119	Growth
	Ethambutol	0.125	0.0050	0.0024	0.0076	Growth
		4	0.0007	-0.0005	0.0020	Stasis
		32	0.0000	-0.0012	0.0012	Stasis
	Amikacin	0.0625	0.0069	0.0043	0.0095	Growth
		1	-0.0131	-0.0289	0.0027	Stasis
		16	-0.0184	-0.0259	-0.0109	Death

**Table 4:** Estimates of the slope of the straight line fitted to data consisting of measured bulk capacitance value (Cb) (dependent variable) as a function of time (t) (independent variable) for various conditions, along with the lower and upper bounds of the 95% confidence interval for *M. bovis BCG*.

*M*. *bovis BCG*						
Sample Type	Antibiotic	Concentration of Antibiotic (μg/mL)	Estimate of Slope	95% CI Lower Bound	95% CI Upper Bound	Inference
**With MNP**	Growth Control	0	0.0427	0.0209	0.0646	Growth
	Pyrazidamine	16	0.0269	0.0125	0.0413	Growth
	Ethambutol	0.125	0.0193	0.0111	0.0275	Growth
		4	-0.0011	-0.0057	0.0036	Stasis
		32	0.0026	-0.0011	0.0063	Stasis
	Amikacin	0.125	0.0192	0.0068	0.0316	Growth
		1	0.0013	-0.0025	0.0052	Stasis
		16	-0.0075	-0.0146	-0.0003	Death
**Without MNP**	Growth Control	0	0.0427	0.0289	0.0564	Growth
	Pyrazidamine	16	0.0426	0.0338	0.0514	Growth
	Ethambutol	0.125	0.0436	0.0368	0.0504	Growth
		4	0.0034	-0.0023	0.0091	Stasis
		32	-0.0004	-0.0061	0.0053	Stasis
	Amikacin	0.125	0.0200	0.0156	0.0244	Growth
		1	0.0000	-0.0041	0.0042	Stasis
		16	-0.0116	-0.0163	-0.0069	Death

## 5. Discussion

In this work, we try to demonstrate “proof of principle” that (a) live mycobacteria can be isolated from sputum using MNPs with high efficiency (almost all the bacteria that survive decontamination) and (b) that the efficacy of candidate drugs on the mycobacteria thus isolated (in suspensions containing MNPs) could be tested in real-time using m-EIS.

As can be seen from our isolation experiments (Table 2 and Fig 6), the decontamination protocol used resulted was largely successful in eliminating “contaminant” Gram-positive and Gram-negative species (less than 0.6% survival for both *M*. *smegmatis* and *M*. *bovis BCG*). However, this was accompanied by significant losses in the number of mycobacteria (only ~70% survival for *M*. *smegmatis* and ~56% survival for *M*. *bovis BCG*). However, it must be noted that the widely used protocol [50], from which our protocols were derived by adjusting the strength of chemicals and time of exposure, was formulated for *M*. *tuberculosis*, a mycobacterium that is hardier than the ones tested by us. Complete (> 5 logs) elimination of contaminant bacteria and > 90% survival (<1 log elimination) of the *M*. *tuberculosis* have been reported for this protocol [51].

More interestingly, virtually all bacteria (both mycobacteria other bacteria) that survive decontamination seem to be collected by the MNPs, with virtually no difference in the loads estimated after decontamination, and after collection using MNPs. It may be noted that these beads were designed to collect *M*. *tuberculosis* cells, and the near-100% collection of other mycobacteria is a better-than-expected outcome. Thus, in a real-world situation (where the decontamination process is likely to lead to complete elimination of contaminants and high survival rates for the mycobacteria), it is very likely that our process is will allow us to collect virtually all the living mycobacteria originally present in the sample into a “pellet”, which can then be re-suspended into a small volume of DST buffer.

The artificial sputum created for this protocol included commensal bacteria typically found in the human sputum samples. We understand that inclusion of human cells such as epithelial cells and mucoid materials (which may be present in the “real world” samples) will make for better approximation of the “real world” samples. Based on our previous experience with m-EIS method [28], where human blood (containing RBCs, WBCs, several proteins and chemicals) in culture media was used for detection of bacteria in the sample, the results obtained by m-EIS demonstrated that the detection was not affected by the presence of these non-proliferating cells/materials. We believe the same will hold true for the presence of epithelial cells and mucoid material. However, the future work will include real-world samples of human sputum with *M tuberculosis* cells with presence of these non-proliferating human cells and materials to determine its effect on our detection modality if any.

Since the main goal of this “proof of principle” study was to ascertain that (a) MIC results could be obtained by bypassing the time-consuming pre-culture of mycobacteria in favor of a rapid pre-concentration step using MNPs, and that (b) the presence of MNPs did not affect the accuracy of the results obtained, standard ATCC strains of *M bovis* and *M smegmatis* were used in the study. “Gold standard” phenotypic DST values for these ATCC strains are well established and available in literature. Thus, the MIC results obtained from the m-EIS method were compared against available MIC values from literature to ensure validity of the results. *M smegmatis* and *M bovis BCG* have known MIC values of 0.5 mg/L [44] and 0.125 mg/L [46] against bactericidal Amikacin respectively, while that of bacteriostatic Ethambutol is 1mg/L [44] and 2 – 4mg/L [48] respectively. Rifampicin is ineffective [45] against *M smegmatis* and pyrazinamide is ineffective against *M bovis BCG* [47]. As can be seen from our m-EIS results (Figs 7 and 8, and Tables 3 and 4), all the results (both with and without MNPs) are consistent with our expectations in the sense that (a) electrical signatures of growth (increase in bulk capacitance) are observed for controls (no drugs), for drugs known to be ineffective, and for bacteriostatic and bactericidal drugs present at concentrations below MIC, (b) electrical signatures of death (decrease in bulk capacitance) are seen for cidal drugs at concentration at and/or above MIC, and (c) electrical signatures corresponding to bacterial stasis (no significant change in bulk capacitance over time) can be seen for bacteriostatic drugs at and above the MIC (and for the bactericidal drug at MIC in the case of *M bovis BCG*). Moreover, similar samples with and without MNPs display similar behavior, thereby laying to rest our apprehensions regarding the potential interference with either the bacterial behavior or the electrical readings.

One major limitation of the current study is that while the two parts of the study (isolation using MNPs and m-EIS) were conducted independently, although care was taken to ensure that the composition of the suspensions subjected to m-EIS was similar to those obtained at the end of the isolation protocol.

Future work will incorporate (a) Real-world sample (human sputum with *M*. *tuberculosis* cells), (b) self-contained and sealable microfluidic cassettes with multiple wells, each containing chosen amounts of candidate drugs pre-loaded into 10μL “wells”, into which suspensions containing growth media, mycobacteria and MNPs can be loaded, and (c) integrated electronics to keep track of multiple wells in parallel. We have previously demonstrated the use of m-EIS to detect mycobacteria in decontaminated sputum samples: both on the basis of detecting their proliferation (a process taking ~36 hours for *M*. *bovis BCG*) [49], and a novel “detection by death” approach [52]. Micro-wells dedicated to implementing these approaches may be incorporated into the same microfluidic cassette to bring together a portable, sensitive, and affordable device to detect *M*. *tuberculosis* in sputum samples, and simultaneously determine the multidrug resistance profile of the pathogen, all within 3 days of sputum collection.

It may be noted that the m-EIS data presented earlier was obtained using suspensions containing mycobacteria at concentrations of ~105 CFU/mL loaded into microchannels in which the volumes of the region between the electrodes was ~10μL. Thus, this implies that 1 condition (candidate drug at a chosen concentration) can be tested with only about 1000 CFUs of mycobacteria. Phenotypic DST for *M*. *tuberculosis* is often performed by testing similar concentrations of mycobacteria (~10 5 CFU/mL) against certain “cutoff” concentrations of candidate drugs [53]. Thus, using only 5000–10,000 cells isolated from a single (or small number of) sputum sample, one can potentially conduct phenotypic drug susceptibility tests against a number of drugs within 3 days (compared to 4 to 6 weeks with current technology).

An important consideration of the research effort has been to achieve DST testing turn-around times comparable to molecular methods like GeneXpert and Line probe Assays (which take 1–2 days [8] while keeping costs comparable to low-cost of traditional phenotypic DST platforms like the MGIT^TM^ and the TREK-ESP^TM^ systems. The latter typically consist of platforms that cost ~$20,000–50,000 (depending on number of samples handled) and per test cost of disposables of < $10 / test. We believe that when our approach is implemented at scale, the cost of the fixed hardware can be kept to around $50,000 and the cost of disposable goods sold (MNPs and microfluidic cassettes pre-spotted with drugs) can be limited to ~$20 / test. The present work is the first step towards demonstrating our ability to achieve rapid turn-around times for DST testing. Moreover, given that in the final product, the readings would be obtained electronically from a sealed microfluidic cassette and the assessment of mycobacterial behavior (growth / death / stasis) can be made without user judgment, this product should be able to overcome the drawback of the MODS technique, and provide a rapid, reliable and relatively inexpensive alternative to current instruments / methods available for phenotypic DST.
